# Variation in contrast-associated acute kidney injury prophylaxis for percutaneous coronary intervention: insights from the Veterans Affairs Clinical Assessment, Reporting, and Tracking (CART) program

**DOI:** 10.1186/s12882-020-01802-z

**Published:** 2020-04-28

**Authors:** Joseph Walker Keach, Maggie A. Stanislawski, Anna E. Barón, Mary E. Plomondon, Paula Langner, Amit Amin, Heather M. Gilmartin, Stephen Waldo, Thomas M. Maddox

**Affiliations:** 1 Department of Medicine, Denver Health Hospital Authority, 601 Broadway MC4000, Denver, CO 80204 USA; 2 Department of Medicine, University of Colorado, Aurora, CO USA; 3 Rocky Mountain Regional VA Medical Center, Aurora, CO USA; 4 Colorado School of Public Health, University of Colorado, Aurora, CO USA; 5 Division of Cardiology, Washington University School of Medicine, St. Louis, MO USA; 6 Healthcare Innovation Lab, BJC HealthCare/Washington University School of Medicine, St. Louis, MO USA

**Keywords:** Prevention, AKI, CA-AKI, PCI, CKD

## Abstract

**Background:**

Contrast-Associated Acute Kidney Injury (CA-AKI) is a serious complication associated with percutaneous coronary intervention (PCI). Patients with chronic kidney disease (CKD) have an elevated risk for developing this complication. Although CA-AKI prophylactic measures are available, the supporting literature is variable and inconsistent for periprocedural hydration and N-acetylcysteine (NAC), but is stronger for contrast minimization.

**Methods:**

We assessed the prevalence and variability of CA-AKI prophylaxis among CKD patients undergoing PCI between October 2007 and September 2015 in any cardiac catheterization laboratory in the VA Healthcare System. Prophylaxis included periprocedural hydration with normal saline or sodium bicarbonate, NAC, and contrast minimization (contrast volume to glomerular filtration rate ratio ≤ 3). Multivariable hierarchical logistic regression models quantified site-specific prophylaxis variability. As secondary analyses, we also assessed CA-AKI prophylaxis measures in all PCI patients regardless of kidney function, periprocedural hydration in patients with comorbid CHF, and temporal trends in CA-AKI prophylaxis.

**Results:**

From 2007 to 2015, 15,729 patients with CKD underwent PCI. 6928 (44.0%) received periprocedural hydration (practice-level median rate 45.3%, interquartile range (IQR) 35.5–56.7), 5107 (32.5%) received NAC (practice-level median rate 28.3%, IQR 22.8–36.9), and 4656 (36.0%) received contrast minimization (practice-level median rate 34.5, IQR 22.6–53.9). After adjustment for patient characteristics, there was significant site variability with a median odds ratio (MOR) of 1.80 (CI 1.56–2.08) for periprocedural hydration, 1.95 (CI 1.66–2.29) for periprocedural hydration or NAC, and 2.68 (CI 2.23–3.15) for contrast minimization. These trends were similar among all patients (with and without CKD) undergoing PCI. Among patients with comorbid CHF (*n* = 5893), 2629 (44.6%) received periprocedural hydration, and overall had less variability in hydration (MOR of 1.56 (CI 1.38–1.76)) compared to patients without comorbid CHF (1.89 (CI 1.65–2.18)). Temporal trend analysis showed a significant and clinically relevant decrease in NAC use (64.1% of cases in 2008 (*N* = 1059), 6.2% of cases in 2015 (*N* = 128, *p* = < 0.0001)) and no significant change in contrast-minimization (*p* = 0.3907).

**Conclusions:**

Among patients with CKD undergoing PCI, there was low utilization and significant site-level variability for periprocedural hydration and NAC independent of patient-specific risk. This low utilization and high variability, however, was also present for contrast minimization, a well-established measure. These findings suggest that a standardized approach to CA-AKI prophylaxis, along with continued development of the evidence base, is needed.

## Relationships with industry

Dr. Waldo receives research support to the Denver Research Institute from Abiomed, Cardiovascular Systems Incorporated and Merck Pharmaceuticals. Dr. Maddox discloses current grant funding from the NIH NCATS (1U24TR002306–01, A National Center for Digital Health Informatics Innovation), current consulting for Creative Educational Concepts, Inc. and Atheneum Partners, and honoraria payments in the past 3 years from Brown University (Sept 2016), Washington State Clinical Outcomes Assessment Program (Oct 2016), Virginia Mason (Oct 2016), University of Utah (May 2017), New York Presbyterian (Sept 2017), Westchester Medical Center (Oct 2017), Sentara Heart Hospital (Dec 2018), and the Henry Ford health system (March 2019). He is currently employed as a cardiologist and director of the Healthcare Innovation Lab at BJC HealthCare/Washington University School of Medicine. In this capacity, he is advising Myia Labs, for which his employer is receiving equity compensation in the company. He is receiving no individual compensation from the company. He is also a compensated director for a New Mexico-based foundation, the J. F Maddox Foundation.

The other authors have no industry relations to disclose.

## Background

Contrast-Associated Acute Kidney Injury (CA-AKI), defined as an absolute increase of serum creatinine ≥0.3 mg/dL or a relative increase ≥50% within 48–72 h of contrast exposure, is a serious complication that may occur after percutaneous coronary intervention (PCI) [1]. CA-AKI is associated with increased morbidity, mortality, hospital length of stay, and healthcare costs [2–5]. Chronic kidney disease (CKD) is the strongest independent risk factor for developing CA-AKI [6]. Unfortunately, effective therapies to reduce the clinical impact of CA-AKI among CKD patients are largely absent. Thus, the primary therapeutic objective is to reduce the likelihood of developing CA-AKI among these patients. Numerous prophylactic interventions have been studied, including periprocedural intravenous hydration with normal saline (NS) or sodium bicarbonate, periprocedural administration of N-Acetylcysteine (NAC), and the minimization of contrast volume [7–11]. The strength of evidence supporting NAC and specific periprocedural hydration protocols is weak, while minimizing procedural contrast volume is much stronger [7–11]. Major society guidelines on CA-AKI prophylaxis in CKD patients are currently lacking or contradictory [12, 13]. We hypothesized that such variability in the literature and guidelines would result in significant site-level practice variation independent of patient-specific factors. We also hypothesized there would be less variability in well-established interventions, such as contrast minimization. With this in mind, we sought to assess the prevalence and variation of CA-AKI prophylactic measures utilized in CKD patients undergoing PCI throughout the largest integrated healthcare system in the United States, the Veterans Affairs (VA) Healthcare System, with hopes of identifying opportunities to improve the use of prophylactic measures and reduce the incidence of CA-AKI.

## Methods

The VA CART program is a national clinical quality initiative for all VA cardiac catheterization laboratories. The program seeks to enhance the quality and safety of invasive cardiovascular procedures throughout the VA [14]. The CART program collects standardized data on all coronary angiograms and percutaneous coronary interventions. Software is embedded in the VA electronic health record (EHR) and allows providers to enter patient and procedural information as part of routine clinical workflow. The CART software was designed using standardized definitions which conform to the definitions and standards of the American College of Cardiology’s National Cardiovascular Data Registry (ACC-NCDR) [15]. Quality checks of the data are periodically conducted for completeness and accuracy [16]. CART data are combined with other VA data sources to create a longitudinal data repository that supports quality assessment and improvement across the integrated healthcare system.

We evaluated the use of periprocedural hydration with normal saline or sodium bicarbonate, periprocedural NAC, and the volume of contrast used during PCI relative to the patient’s GFR among patients with CKD undergoing PCI between October 1st 2007 and September 30th 2015 at any VA cardiac catheterization laboratory. Prior studies have indicated that ratios of contrast volume relative to GFR < 3 are associated with lower CA-AKI risk [10]. CKD was defined as a glomerular filtration rate (GFR) of 15-59 mL/min. The use of periprocedural hydration was determined from in-lab records and/or inpatient medications within 48 h of the procedure and included the administration of normal saline (NS) or intravenous sodium bicarbonate. Doses of periprocedural hydration < 100 mL were excluded to avoid mistaking saline flushes and other small saline applications for CA-AKI pre-hydration. The median volume of preprocedural hydration administered using this approach was 1000 ml (IQR: 500–1000 ml). The use of NAC was determined from in-lab records and/or outpatient prescription data, including prescriptions for < 3 days of NAC given within 30 days of the procedure. The PCI providers recorded contrast volume administration in the EHR. GFR levels were calculated using the Modification of Diet in Renal Disease (MDRD) equation, using the most recent serum creatinine value recorded within 30 days of the PCI [17]. Patients were excluded for currently receiving hemodialysis, missing pre-procedural GFR assessment, missing longitudinal follow-up data, undergoing repeat PCI, or receiving PCI at a facility that performed < 50 PCIs over the time frame of the study.

We assessed patient demographic and clinical risk factors, including age, race (white, black, other), sex, presence of congestive heart failure (CHF), GFR, and history of diabetes, from the EHR using standard definitions [15]. Lesion risk (defined as high or non-high) and number of stents were determined by data entered into the CART application by physicians. Annual PCI volume was assessed using CART data.

We compared demographic, clinical, and CA-AKI prophylaxis characteristics of CKD patients undergoing PCI by catheterization laboratory rates of periprocedural hydration or NAC use, stratified into quartiles. We used chi-squared tests for categorical variables and Mann–Whitney Wilcoxon tests for continuous variables. We then calculated unadjusted rates of 1) Periprocedural hydration, 2) NAC use, 3) periprocedural hydration or NAC use, and 4) contrast: GFR ≤ 3. Median site-level rates with interquartile ranges were calculated.

Next, to evaluate the variation in use of site-level CA-AKI prophylaxis measures independent of patient CA-AKI risk, we calculated adjusted rates of 1) periprocedural hydration, 2) periprocedural hydration or NAC, and 3) contrast: GFR ≤ 3. We did not model the outcome of NAC due to instability in statistical models caused by the wide variation in NAC use across sites with very small numbers at many sites. For the analysis of each prophylaxis measure, catheterization laboratories were excluded if the prophylactic measure was used infrequently (< 20 times over the study period) to avoid unstable estimates. For the contrast: GFR ratio analyses, PCI patients who had missing contrast volume information were excluded.

We used hierarchical regression models with VA catheterization laboratory sites as a random intercept to adjust for differences in lab case mix and to account for the clustering of patients by lab, similar to the methodology used in calculating hospital-level readmission data by the Centers for Medicare and Medicaid [18]. Based on prior literature and a priori clinical knowledge, we adjusted for the following patient level characteristics associated with CA-AKI: age, race, sex, CHF, GFR, diabetes, lesion risk (at least 1 high risk vs all non-high risk), number of stents, and year of PCI, as well as site-level annual PCI volume. Lesion risk and number of stents were included as surrogates for PCI complexity. For binary outcomes, we used logistic regression models to calculate the predicted and expected probability of each prevention measure at each lab and then multiplied this ratio by the mean rate across sites to get the standardized site-level rate [19]. We estimated the 95% confidence interval using bootstrap sampling. We considered the lab proportion as significantly different from average when the 95% confidence interval did not include the system-wide mean.

We used the median odds ratio (MOR) to describe the level of variation between sites, independent of patient factors. The MOR can be interpreted as the odds that 2 patients with identical patient-level covariates from 2 randomly chosen sites will receive similar treatments, or in our case, prophylaxis. An MOR of 1.0 indicates that no variation exists between sites, and identical patients would receive identical treatment at different sites. The MOR is always ≥1. For example, an MOR of 1.5 indicates a 50% likelihood that a similar patient would receive different prophylactic measures at 2 different sites. It provides an estimate of the effect of the catheterization laboratory on the outcome, much as the odds ratio (OR) estimates the effect of patient factors on the outcome. Based on previous literature, an MOR > 1.2 indicates moderate site-level variation [20].

We also performed several secondary analyses. First, we expanded the cohort to all patients undergoing PCI, regardless of kidney function. Methods for this analysis of all patients were identical to those used for the primary analysis.

Second, to evaluate the potential effect of comorbid CHF on the volume of IV fluid administered for prophylaxis, we stratified the cohort by presence of CHF. We then performed hierarchical regression models of periprocedural hydration, identical to those used for the primary analysis, to estimate use, and variation in use, within each stratum.

Finally, to explore the temporal changes in prophylaxis, a linear trend test was applied to unadjusted rates of use of each measure by fiscal year.

All statistical analyses were performed by the CART Program Analytic Center using SAS version 9.4 (SAS Institute Inc., Cary, North Carolina) and R 3.4.1 (R Core Team (2019). The Colorado Multiple Institutional Review Board approved the study.

## Results

**Fig. 1:**
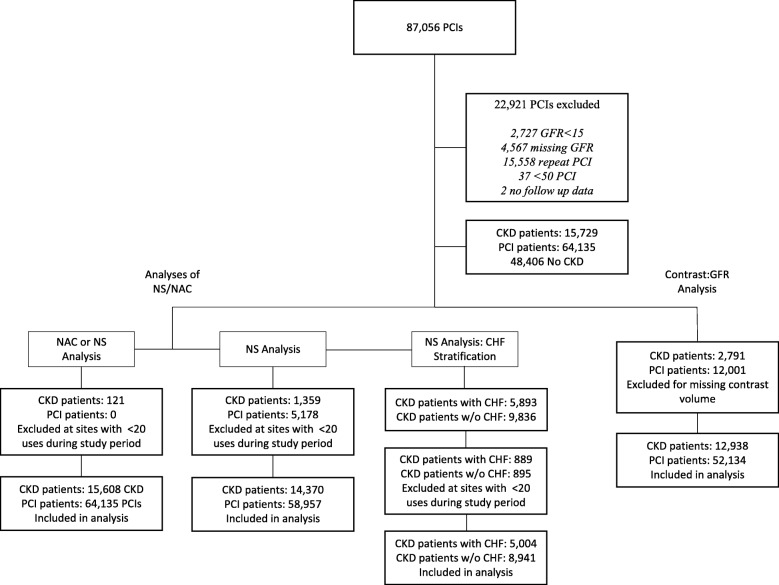
Primary cohort flow diagram

Between 2007 and 2015, 87,056 PCIs were performed across the VA integrated healthcare system. As shown in Fig. 1, PCIs were excluded if patients were currently receiving hemodialysis (*N* = 2727), missing pre-procedural GFR assessment (*N* = 4567), without longitudinal follow-up data (*N* = 2), undergoing repeat PCI (*N* = 15,588), sites with < 50 PCIs over the time frame of the study (*N* = 37), and or had normal kidney function (*N* = 48,406). Thus, the cohort for our primary analysis included 15,729 patients with CKD undergoing PCI at 64 unique sites.

**Fig. 2:**
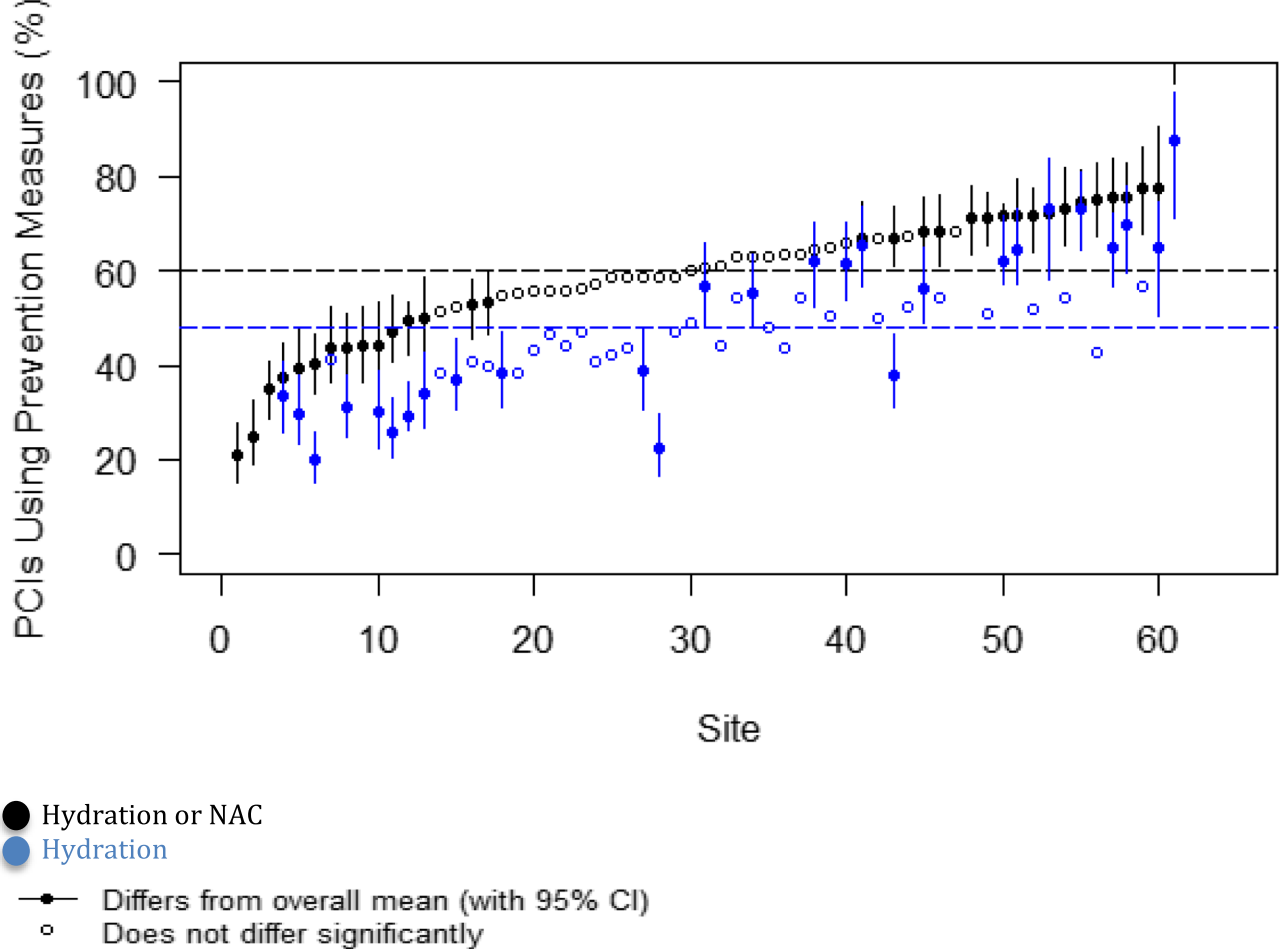
Plot of risk-adjusted, site-level rates of hydration and NAC prophylaxis in PCI patients with CKD. The estimates are shown with 95% confidence intervals (CIs) for those that differ significantly from the system-wide average. Estimates are risk-adjusted for the following: CHF, age, sex, race (white, black, other), GFR, diabetes, lesion risk (at least 1 high vs all non-high), number of stents, year of PCI, and annual hospital PCI volume

**Fig. 3:**
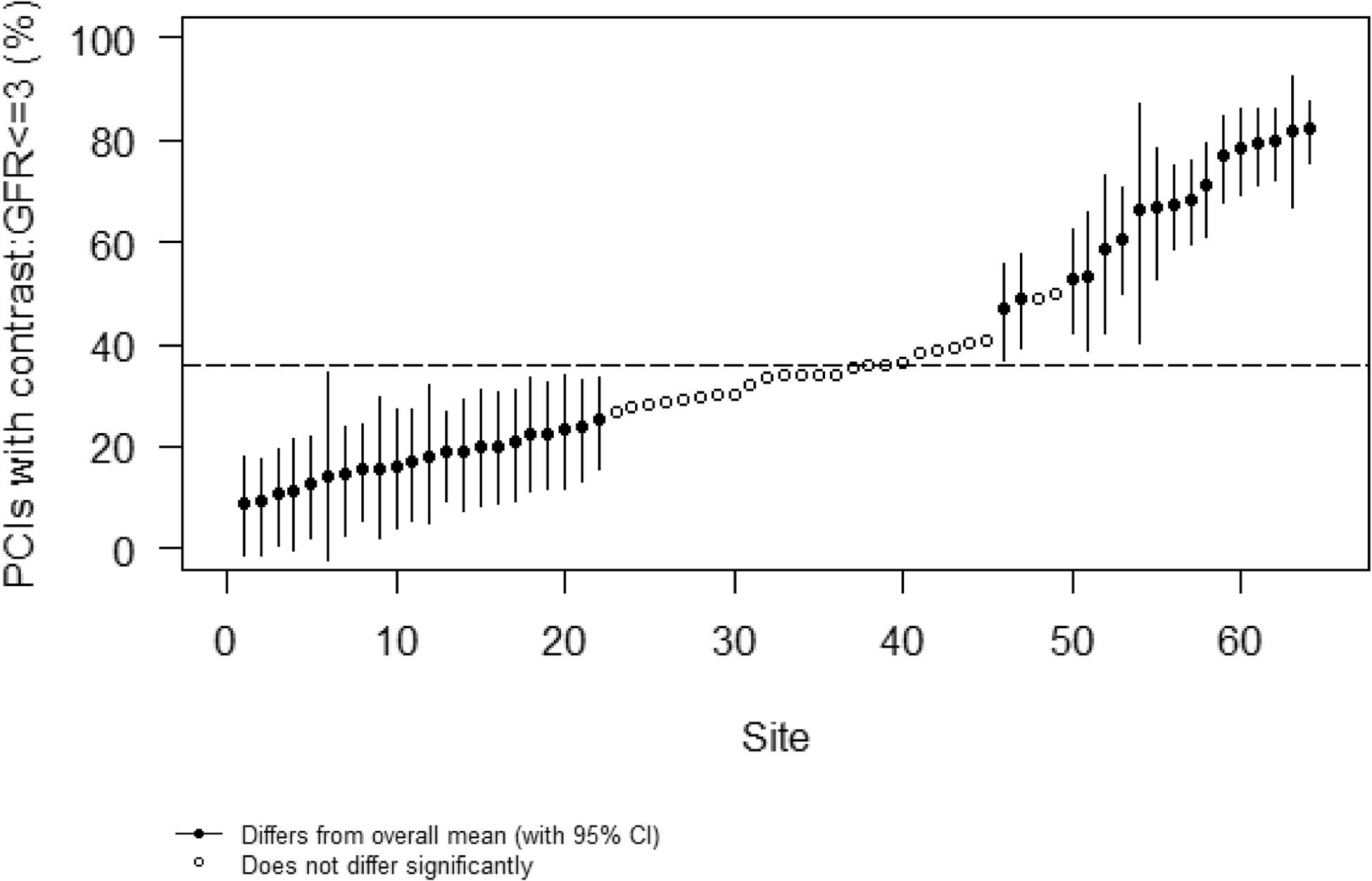
Plot of risk-adjusted site-level PCIs with contrast to GFR ratios ≤3 among PCI patients with CKD. The estimates are shown with 95% confidence intervals (CIs) for those that differ significantly from the system-wide average. Estimates are risk-adjusted for the following: CHF, age, sex, race (white, black, other), GFR, diabetes, lesion risk (at least 1 high vs all non-high), number of stents, year of PCI, and annual hospital PCI volume

Table 1 shows the characteristics of the primary CKD cohort, both overall and arranged by quartile of increasing unadjusted rates of periprocedural hydration or NAC use. Among these 15,729 patients, 6928 (44.0%) received periprocedural hydration (practice-level median rate 45.3%, interquartile range (IQR) 35.5–56.7), 5107 (32.5%) received NAC (practice-level median rate 28.3%, IQR 22.8–36.9), and 9436 (60.0%) received periprocedural hydration or NAC (practice-level median rate 62.8, IQR 50.7–72.4). The median contrast: GFR ratio was 3.6, (IQR 2.5–5.1) and 4656 (36.0%) patients received contrast minimization (contrast: GFR ratio ≤ 3, practice-level median rate 34.5, IQR 22.6–53.9). After adjusting for patient-specific risk factors, the MOR for only periprocedural hydration was 1.80 (CI 1.59–2.08), for periprocedural hydration or NAC was 1.95 (confidence interval [CI] 1.66–2.29), and contrast: GFR ratio ≤ 3 was 2.68 (CI 2.23–3.15) (Table 2; Figs. 2 and 3). The intracluster correlation coefficient for periprocedural hydration was 0.07, for hydration or NAC, 0.13, and for contrast minimization, 0.18.

**Table 1:** Demographic and clinical characteristics of patients with CKD receiving PCI arranged by site quartile of unadjusted rates of periprocedural hydration or NAC use

Variable	Total (*N* = 15,729)	QUARTILE 1 (*N* = 3507)	QUARTILE 2 (*N* = 4108)	QUARTILE 3 (*N* = 4511)	QUARTILE 4 (*N* = 3603)	*p*-value
Demographics						
Age (Median (IQR))	68.6 (63.6–76.9)	68.8 (63.5–76.8)	67.7 (63.1–75.6)	68.6 (63.4–76.9)	70.0 (64.3–78.7)	<.0001
Male	15,429 (98.1%)	3439 (98.1%)	4022 (97.9%)	4438 (98.4%)	3530 (98.0%)	0.38
Race						
White	13,613 (86.5%)	2997 (85.5%)	3525 (85.8%)	3919 (86.9%)	3172 (88.0%)	<.0001
Black	1799 (11.4%)	421 (12.0%)	525 (12.8%)	515 (11.4%)	338 (9.4%)	
Other	317 (2.0%)	89 (2.5%)	58 (1.4%)	77 (1.7%)	93 (2.6%)	
Cormorbidities						
Hypertension	14,973 (95.2%)	3332 (95.0%)	3953 (96.2%)	4242 (94.0%)	3446 (95.6%)	<.0001
Hyperlipidemia	14,413 (91.6%)	3146 (89.7%)	3855 (93.8%)	4077 (90.4%)	3335 (92.6%)	<.0001
Diabetes	9127 (58.0%)	2060 (58.7%)	2407 (58.6%)	2552 (56.6%)	2108 (58.5%)	0.14
Tobacco Use	9124 (58.0%)	1916 (54.6%)	2599 (63.3%)	2339 (51.9%)	2270 (63.0%)	<.0001
Prior MI	6728 (42.8%)	1503 (42.9%)	1804 (43.9%)	1860 (41.2%)	1561 (43.3%)	0.071
Prior PCI	6018 (38.3%)	1328 (37.9%)	1602 (39.0%)	1743 (38.6%)	1345 (37.3%)	0.43
Congestive Heart Failure	5893 (37.5%)	1330 (37.9%)	1503 (36.6%)	1711 (37.9%)	1349 (37.4%)	0.56
Cerebrovascular Disease	3803 (24.2%)	881 (25.1%)	982 (23.9%)	1034 (22.9%)	906 (25.1%)	0.055
Peripheral Arterial Disease	4451 (28.3%)	970 (27.7%)	1152 (28.0%)	1220 (27.0%)	1109 (30.8%)	0.0016
Hx of CKD Dx	8707 (55.4%)	1876 (53.5%)	2319 (56.5%)	2308 (51.2%)	2204 (61.2%)	<.0001
Chronic Obstructive Pulmonary Disease	4136 (26.3%)	930 (26.5%)	1035 (25.2%)	1229 (27.2%)	942 (26.1%)	0.19
BMI (Median (IQR))	29.9 (26.4–34.0)	30.0 (26.6–34.1)	30.1 (26.6–34.2)	29.7 (26.1–33.7)	29.9 (26.4–34.1)	0.0048
Chronic Depression	4407 (28.0%)	1004 (28.6%)	1263 (30.7%)	1213 (26.9%)	927 (25.7%)	<.0001
Sleep Apnea	3596 (22.9%)	765 (21.8%)	1008 (24.5%)	993 (22.0%)	830 (23.0%)	0.014
Baseline Cholesterol (Median (IQR))	157.7 (135.3–185.0)	161.5 (138.0–189.0)	160.1 (137.2–188.0)	155.2 (133.0–183.0)	154.0 (133.0–181.0)	<.0001
Baseline LDL (Median (IQR))	85.8 (67.7–108.4)	86.3 (68.3–110.0)	87.1 (68.0–109.2)	86.0 (68.0–108.3)	83.3 (66.0–106.0)	<.0001
Baseline HDL (Median (IQR))	36.7 (31.0–43.5)	36.6 (31.0–43.6)	36.5 (31.0–43.0)	36.3 (31.0–43.0)	37.1 (31.7–44.3)	0.0006
CKD class						
3a	11,029 (70.1%)	2404 (68.5%)	2958 (72.0%)	3140 (69.6%)	2527 (70.1%)	0.02
3b	3927 (25.0%)	915 (26.1%)	981 (23.9%)	1135 (25.2%)	896 (24.9%)	
4	773 (4.9%)	188 (5.4%)	169 (4.1%)	236 (5.2%)	180 (5.0%)	
Baseline GFR (Median (IQR))	50.4 (43.0–55.7)	50.0 (42.4–55.1)	50.9 (43.8–56.0)	50.6 (43.0–56.0)	50.2 (42.9–55.3)	0.0012
Procedural Details						
PCI Status						
Elective	9617 (61.1%)	2275 (64.9%)	2744 (66.8%)	2545 (56.4%)	2053 (57.0%)	<.0001
Emergent/Urgent	6034 (38.4%)	1220 (34.8%)	1329 (32.4%)	1947 (43.2%)	1538 (42.7%)	
Missing	78 (0.50%)	12 (0.3%)	35 (0.9%)	19 (0.4%)	12 (0.3%)	
PCI Indication						
STEMI	1094 (7.0%)	263 (7.5%)	252 (6.1%)	336 (7.4%)	243 (6.7%)	<.00
NSTEMI	3515 (22.3%)	756 (21.6%)	789 (19.2%)	1074 (23.8%)	896 (24.9%)	
Unstable Angina	3516 (22.4%)	682 (19.4%)	1154 (28.1%)	918 (20.4%)	762 (21.1%)	
ACS	182 (1.2%)	39 (1.1%)	44 (1.1%)	74 (1.6%)	25 (0.7%)	
Stable Angina	4693 (29.8%)	1218 (34.7%)	1119 (27.2%)	1285 (28.5%)	1071 (29.7%)	
Other/Missing	2729 (17.4%)	549 (15.7%)	750 (18.3%)	824 (18.3%)	606 (16.8%)	
At least 1 lesion of high risk	6586 (41.9%)	1578 (45.0%)	1535 (37.4%)	2056 (45.6%)	1417 (39.3%)	<.0001
Number of stents (Median (IQR))	1.0 (1.0–1.0)	1.0 (1.0–1.0)	1.0 (1.0–1.0)	1.0 (1.0–1.0)	1.0 (1.0–1.0)	0.64
CA-AKI Prevention Measures						
Mutually Exclusive Categories of Preventions						
None	6293 (40.0%)	2028 (57.8%)	1778 (43.3%)	1537 (34.1%)	950 (26.4%)	<.0001
Only NS	4329 (27.5%)	533 (15.2%)	1004 (24.4%)	1464 (32.5%)	1328 (36.9%)	
Only NAC	2508 (15.9%)	691 (19.7%)	604 (14.7%)	572 (12.7%)	641 (17.8%)	
NAC and Saline	2599 (16.5%)	255 (7.3%)	722 (17.6%)	938 (20.8%)	684 (19.0%)	
Any NAC	5107 (32.5%)	946 (27.0%)	1326 (32.3%)	1510 (33.5%)	1325 (36.8%)	<.0001
Any Hydration	6928 (44.0%)	788 (22.5%)	1726 (42.0%)	2402 (53.2%)	2012 (55.8%)	<.0001
Hydration or NAC	9436 (60.0%)	1479 (42.2%)	2330 (56.7%)	2974 (65.9%)	2653 (73.6%)	<.0001
Contrast use	(*N* = 12,938)	(*N* = 2988)	(*N* = 3096)	(*N* = 3720)	(*N* = 3134)	
Contrast (Median (IQR))	175.0 (120.0–246.0)	165.0 (100.0–230.0)	175.0 (120.0–240.0)	190.0 (132.0–260.0)	165.0 (105.0–235.0)	<.0001
Contrast:GFR (Median (IQR))	3.6 (2.5–5.1)	3.4 (2.2–5.0)	3.6 (2.5–5.1)	4.0 (2.8–5.4)	3.5 (2.3–4.9)	<.0001
Contrast:GFR ≤ 3	4656 (36.0%)	1206 (40.4%)	1136 (36.7%)	1081 (29.1%)	1233 (39.3%)	<.0001

**Table 2:** Risk-Adjusted Median Odds Ratio for CA-AKI prophylaxis measures in PCI patients with CKD and all PCI patients (CKD or normal kidney function)

	CKD Patients		All Patients	
	MOR	95% CI	MOR	95% CI
Hydration	1.80	1.59–2.08	1.96	1.71–2.24
Hydration or NAC	1.95	1.66–2.29	2.15	1.82–2.53
Contrast:GFR Ratio ≤ 3	2.68	2.23–3.15	2.75	2.26–3.28

Estimates risk-adjusted for the following: CHF, age, sex, race (white, black, other), GFR, diabetes, lesion risk (at least 1 high vs all non-high), # of stents, year of PCI, and annual hospital PCI volume

For our secondary analysis of all PCI patients, we included all patients undergoing PCI during the study period regardless of kidney function. Among this cohort of 64,135 PCI patients, 22,888 (35.7%) received periprocedural hydration, 8919 (13.9%) received NAC, and 27,595 (43%) received periprocedural hydration or NAC. The median contrast volume to GFR ratio was 2.5 (IQR 1.6–3.7) and 32,503 (62.3%) patients had a contrast: GFR ratio < = 3. After adjustment, the MOR for periprocedural hydration was 1.96 (CI 1.71–2.24), and for periprocedural hydration or NAC use was 2.15 (CI 1.82–2.53). The MOR for a contrast: GFR ratio ≤ 3 was 2.75 (CI 2.26–3.28) (Table 2; Supplemental Figs. 1 and 2).

For our secondary analysis of comorbid CHF and periprocedural hydration, 5893 (37.5%) patients from our primary cohort had CHF (9836 had CKD but not CHF). 2629 (44.61%) patients with CHF received periprocedural hydration, while 4299 (43.71%) of those without CHF received hydration. After adjusting for patient-specific risk factors, the MOR for any periprocedural hydration among patients with CHF was 1.56 (1.38–1.76). For those without CHF, the MOR was 1.89 (1.65–2.18) (Supplemental Fig. 3).

Finally, for our secondary analysis of temporal prophylaxis trends, there was a significant and clinically relevant decrease in NAC use over the study period, with NAC being used in 64.1% of cases in 2008 (*N* = 12,059), and only 6.2% of cases in 2015 (*N* = 128, *p* = < 0.0001) (Supplemental Tables 1a, b, c). Contrast minimization appeared to stay constant with no significant trend change over the study period (*p* = 0.3907).

## Discussion

Our analysis of CA-AKI prophylaxis among patients with CKD undergoing PCI in the national VA healthcare system demonstrated low utilization of commonly employed prophylactic measures, as well as a high level of variability in practice patterns, independent of patient-specific risk. This low utilization and high variability were present for well-established, as well as less established, prophylactic measures and similar trends were noted in PCI patients with and without CKD. We expected there would be less variability in CA-AKI prophylaxis in the highest risk population, patients with CKD, compared to the general PCI population. We found, however, that high levels of variability persisted among CKD patients, with contrast minimization even more variable in this population.

Among patients with CKD, periprocedural hydration and NAC, both poorly-established interventions, were administered in a little over half of procedures, and had an MOR of 1.80, indicating that identical patients would have roughly 2-fold greater odds of receiving hydration or NAC by being treated in one facility compared to another. However, the well-established measure of contrast minimization had even higher variability by site (MOR 2.68). This demonstrates that identical patients with CKD have almost 3-fold greater odds of receiving high volumes of contrast in 1 cardiac catheterization laboratory compared to another.

Among patients with comorbid CHF, we found that patients with CKD and comorbid CHF received periprocedural hydration at a similar frequency (44.61% vs 43.71%). Furthermore, after risk adjustment, there was less variability in periprocedural hydration of patients with CHF than those without (MOR 1.56 vs 1.89, respectively). This is likely related to provider preference and avoidance of potential volume overload in patients at high risk for this complication (patients with impaired cardiac and renal function).

Our study evaluated 8 years of CA-AKI prophylaxis practice patterns. During this time significant research was published and subsequent guidelines updated. We observed a statistically significant and clinically relevant decrease in the use of NAC prophylaxis over the time course of our study, falling from 64.1% of cases in 2008 to only 6.2% in 2015. This is likely related to high-quality research demonstrating the lack of benefit of NAC for CA-AKI prophylaxis, and the updated 2011 ACC/AHA/SCAI guidelines recommended against the use of NAC [12]. Of note, the well-established prophylactic measure of contrast-minimization remained relatively low and constant throughout our study period. Contrast-minimization was on average utilized only 36.0% of the time and ranged from 33.6 to 38.9% with no significant increase in utilization over time.

To our knowledge, only a small number of studies have investigated the prevalence of CA-AKI prophylactic measure utilization, none of which analyzed contrast minimization strategies, or PCI and CKD specifically [21–24]. A 2008 single-center study by Weisbord et al. prospectively identified 660 patients with CKD undergoing intravenous and intra-arterial contrasted studies and showed that periprocedural fluids were administered in 40% of patients and NAC was administered in 39.2% [23]. A 2014 study by Lee et al. retrospectively analyzed the incidence of CA-AKI and prevalence of prophylactic measure utilization with intravenous contrasted computed tomography (CT) scans. They identified 101,487 patients with 140,838 contrasted CT scans and found the incidence of prophylactic medication utilization to be 28.6%, mostly driven by NS use (26%) [24].

Compared to these, our study specifically analyzed the prevalence of CA-AKI prophylaxis surrounding PCI in patients with and without CKD, including contrast minimization. Our finding of periprocedural hydration or NAC administration in 43% of cases (patients with and without CKD) is within range of prior literature. In addition, we add to this literature with our demonstration of a high level of patient-adjusted variability in CA-AKI prophylaxis among high-risk patients, including contrast minimization, and accounting for patient-level risk factors.

The significant variation in CA-AKI prophylaxis likely reflects the ambiguity of the underlying data, particularly in light of recent randomized clinical trials. A study by Nijssen et al. demonstrated no benefit when comparing periprocedural hydration with NS to no intravenous hydration at all [25]. The PRESERVE trial randomly assigned 5177 patients to receive periprocedural hydration with intravenous sodium bicarbonate or NS, as well as either NAC or placebo, in a 2-by-2 factorial design. The study demonstrated no benefit of sodium bicarbonate compared to NS, as well as no benefit of NAC when compared to placebo [26].

Conflicting major society guidelines likely compound the effect of the contradictory literature. The 2011 ACCF/AHA/SCAI guidelines for PCI recommend periprocedural intravenous hydration without a specific protocol for volume or timing of hydration and recommend against NAC [12]. The 2012 KDIGO guidelines recommend periprocedural intravenous hydration without specific protocols for volume or timing of hydration, however they do recommend the use of NAC [13]. Accordingly, this inconsistency in the hydration and NAC guidelines makes our findings of high variability in their utilization somewhat expected.

In contrast to these inconsistencies, there is broad consensus and guideline support for minimizing contrast during PCI, and numerous studies have demonstrated a contrast: GFR volume < 3 is associated with lower rates of CA-AKI [10]. This makes our findings of high variability in contrast minimization, both in our overall population and among those with CKD, very surprising.

Our study had several limitations, the first being that our outcomes were determined from analyzing the electronic health record, and some data may have been incompletely or inaccurately recorded, with accuracy varying by site. This could have resulted in inaccurate measurement of prophylactic measures. Second, the results may be subject to confounding variables not accounted for by our statistical models. We adjusted for numerous variables associated with CA-AKI, however, there could be unaccounted for confounders that influenced our results. Third, we used an arbitrary volume of > 100 mL periprocedural hydration as a cutoff for receiving prophylactic hydration. However, the most commonly used NS hydration protocols (3–4 mL/kg per hour 4 h before and 4 h after contrast administration, or 1 mL/kg per hour 12 h before and 12 h after) would administer 1.5–2.0 l of NS for a typical 70 kg patient; thus a cutoff of 100 mL of NS likely over estimates the rate of prophylactic NS administration [25]. Fourth, there are numerous other CA-AKI prophylactic strategies available, including statin therapy and ascorbic acid, which were not evaluated in this study. However, as periprocedural hydration, NAC, and contrast minimization have the strongest body of evidence supporting their use, we believe they are adequate surrogates. Fifth, there is ongoing debate in the literature on the prevalence and nature of CA-AKI, with some evidence suggesting the prevalence is lower than originally thought [27]. This underlying confusion, however, likely leads to some of the variation we identified, and until this condition is better understood practice variation will continue. Sixth, we did not capture whether certain nephrotoxic home medications were held or continued (diuretics, NSAIDs, ACE/ARB/RAAS inhibitors, etc.). This could have potentially informed our investigation further on CA-AKI prophylactic provider behaviors. Seventh, we did not adjust for elective vs. urgent/emergent procedure type, nor the presence or absence of cardiogenic shock. We did, however, adjust for the presence or absence of CHF as well as lesion risk and number of stents, which we felt were adequate surrogates. Eighth, we acknowledge that contrast minimization may not have been possible in certain cases and variability in this may not necessarily reflect provider non-use of the intervention.

## Conclusion

In conclusion, our study demonstrated a low overall prevalence of CA-AKI prophylaxis, and a high level of site variation in VA catheterization laboratories for both patients with CKD and the overall PCI population. This variability was present with both established methods, such as contrast minimization, and contestable methods, such as hydration or NAC administration. There are likely multiple factors causing this variability, including conflicting literature, vague and contradictory guidelines, and provider preference. Proven methods to prevent CA-AKI, such as contrast minimization, should be standardized across the healthcare system, while further research should be pursued to establish other effective measures to prevent CA-AKI, and prospectively evaluate the influence of standardized CA-AKI prophylaxis protocols on patient outcomes.

## Supplementary information

**Additional file 1: Table S1a, b, and c** Temporal Trends in CA-AKI prophylaxis utilization

**Additional file 2: Figure S1** Plot of risk-adjusted, site-level rates of hydration and NAC prophylaxis among all PCI patients (with and without CKD). The estimates are shown with 95% confidence intervals (CIs) for those that differ significantly from the system-wide average. Estimates are risk-adjusted for the following: CHF, age, sex, race (white, black, other), GFR, diabetes, lesion risk (at least 1 high vs all non-high), number of stents, year of PCI, and annual hospital PCI volume. **Figure S2** Plot of risk-adjusted, site-level PCIs with contrast to GFR ratios ≤3 among all PCI patients (with and without CKD). The estimates are shown with 95% confidence intervals (CIs) for those that differ significantly from the system-wide average. Estimates are risk-adjusted for the following: CHF, age, sex, race (white, black, other), GFR, diabetes, lesion risk (at least 1 high vs all non-high), number of stents, year of PCI, and annual hospital PCI volume. **Figure S3** Plot of site-level saline use among CKD patients, stratified by CHF. The estimates are shown with 95% confidence intervals (CIs) for those that differ significantly from the system-wide average. Estimates are risk-adjusted for the following: age, sex, race (white, black, other), GFR, diabetes, lesion risk (at least 1 high vs all non-high), number of stents, year of PCI, and annual hospital PCI volume.

## Data Availability

The datasets used and/or analyzed during the current study are available from the corresponding author on reasonable request.

## Abbreviations

CA-AKI: Contrast-Associated Acute Kidney Injury
GFR: glomerular filtration rate
PCI: percutaneous coronary intervention
NAC: N-Acetylcysteine
CKD: chronic kidney disease
MOR: median odds ratio
