# Supplementary material for: Variation in contrast-associated acute kidney injury prophylaxis for percutaneous coronary intervention: insights from the Veterans Affairs Clinical Assessment, Reporting, and Tracking (CART) program
Source: BMC Nephrol. 2020 Apr 28;21:150. doi: 10.1186/s12882-020-01802-z (PMC7189455; doi:10.1186/s12882-020-01802-z)
Supplement: Supplementary file 1 — Additional file 1: Table S1a, b, and c Temporal Trends in CA-AKI prophylaxis utilization [file 12882_2020_1802_MOESM1_ESM.docx]

**Supplemental Tables 1a, 1b, and 1c: Temporal Trends in CA-AKI prophylaxis utilization**

**Supplemental Table 1a**

| **CA-AKI Prevention Measures (N = 15,729)** | | | | |
| --- | --- | --- | --- | --- |
| **Fiscal Year** | **N** | **Hydration (N (%))** | **NAC (N (%))** | **Hydration or NAC (N (%))** |
| 2008 | 1,651 | 706 (42.76) | 12,059 (64.14) | 1,257 (76.14) |
| 2009 | 1,767 | 808 (45.73) | 1,053 (59.59) | 1,309 (74.08) |
| 2010 | 2,040 | 934 (45.78) | 1,222 (59.9) | 1,521 (74.56) |
| 2011 | 2,101 | 954 (45.41) | 836 (39.79) | 1,364 (64.92) |
| 2012 | 2,035 | 950 (46.68) | 457 (22.46) | 1,159 (56.95) |
| 2013 | 1,969 | 904 (45.91) | 200 (10.16) | 1,007 (51.14) |
| 2014 | 2,097 | 855 (40.77) | 152 (7.25) | 935 (44.59) |
| 2015 | 2,069 | 817 (39.49) | 128 (6.19) | 884 (42.73) |

**Supplemental Table 1b**

| **Contrast Use (N = 12,938)** | | |
| --- | --- | --- |
| **Fiscal Year** | **N** | **Contrast:GFR <= 3 (N (%))** |
| 2008 | 1,181 | 454 (38.44) |
| 2009 | 1,261 | 491 (38.94) |
| 2010 | 1,592 | 555 (34.86) |
| 2011 | 1,772 | 595 (33.58) |
| 2012 | 1,775 | 629 (35.44) |
| 2013 | 1,704 | 603 (35.39) |
| 2014 | 1,841 | 650 (35.31) |
| 2015 | 1,812 | 679 (37.47) |

**Supplemental Table 1c**

| **Cochran-Armitage Trend Test** | |
| --- | --- |
| **Prevention Measures** | **p** |
| Hydration *** | 0.0005 |
| NAC | <.0001 |
| Hydration or NAC | <.0001 |
| Contrast:GFR <= 3 | 0.3907 |

*** Although the temporal trend in hydration is significant (p=0.0005), the odds of use decreased only slightly on average from one year to the next (OR=1.025, 95% CI: 1.011, 1.040).
