# Supplementary material for: Variation in contrast-associated acute kidney injury prophylaxis for percutaneous coronary intervention: insights from the Veterans Affairs Clinical Assessment, Reporting, and Tracking (CART) program
Source: BMC Nephrol. 2020 Apr 28;21:150. doi: 10.1186/s12882-020-01802-z (PMC7189455; doi:10.1186/s12882-020-01802-z)
Supplement: Supplementary file 2 — Additional file 2: Figure S1 Plot of risk-adjusted, site-level rates of hydration and NAC prophylaxis among all PCI patients (with and without CKD). The estimates are shown with 95% confidence intervals (CIs) for those that differ significantly from the system-wide average. Estimates are risk-adjusted for the following: CHF, age, sex, race (white, black, other), GFR, diabetes, lesion risk (at least 1 high vs all non-high), number of stents, year of PCI, and annual hospital PCI volume. Figure S2 Plot of risk-adjusted, site-level PCIs with contrast to GFR ratios ≤3 among all PCI patients (with and without CKD). The estimates are shown with 95% confidence intervals (CIs) for those that differ significantly from the system-wide average. Estimates are risk-adjusted for the following: CHF, age, sex, race (white, black, other), GFR, diabetes, lesion risk (at least 1 high vs all non-high), number of stents, year of PCI, and annual hospital PCI volume. Figure S3 Plot of site-level saline use among CKD patients, stratified by CHF. The estimates are shown with 95% confidence intervals (CIs) for those that differ significantly from the system-wide average. Estimates are risk-adjusted for the following: age, sex, race (white, black, other), GFR, diabetes, lesion risk (at least 1 high vs all non-high), number of stents, year of PCI, and annual hospital PCI volume. [file 12882_2020_1802_MOESM2_ESM.docx]

Supplemental Figure 1: Plot of risk-adjusted, site-level rates of hydration and NAC prophylaxis among all PCI patients (with and without CKD)


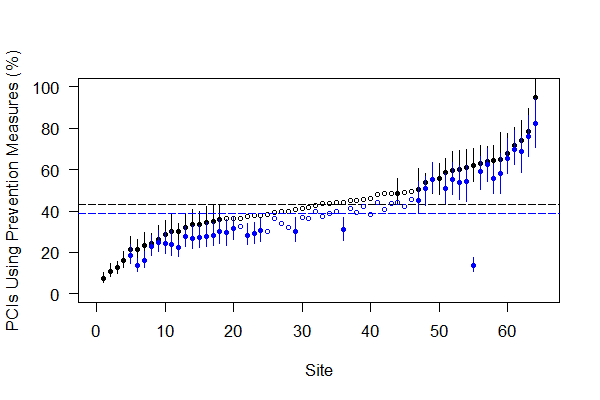


Hydration or NAC

Hydration


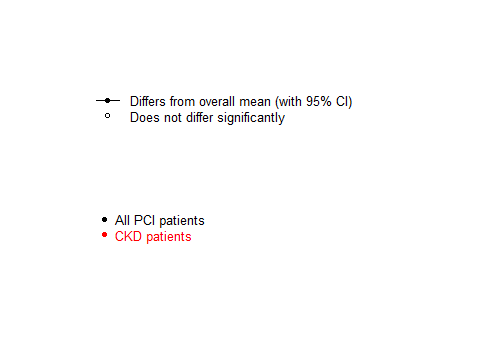


*The estimates are shown with 95% confidence intervals (CIs) for those that differ significantly from the system-wide average. Estimates are risk-adjusted for the following: CHF, age, sex, race (white, black, other), GFR, diabetes, lesion risk (at least 1 high vs all non-high), number of stents, year of PCI, and annual hospital PCI volume.*

Supplemental Figure 2: Plot of risk-adjusted, site-level PCIs with contrast to GFR ratios ≤ 3 among all PCI patients (with and without CKD)


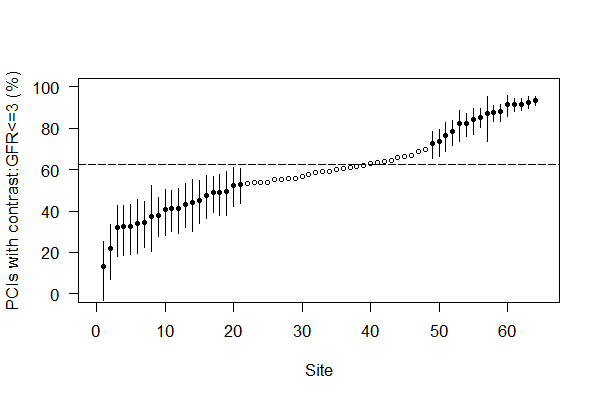


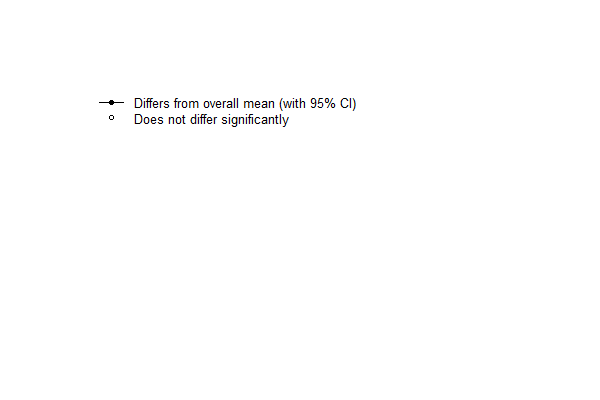


*The estimates are shown with 95% confidence intervals (CIs) for those that differ significantly from the system-wide average. Estimates are risk-adjusted for the following: CHF, age, sex, race (white, black, other), GFR, diabetes, lesion risk (at least 1 high vs all non-high), number of stents, year of PCI, and annual hospital PCI volume.*

Supplemental Figure 3: Plot of site-level saline use among CKD patients, stratified by CHF


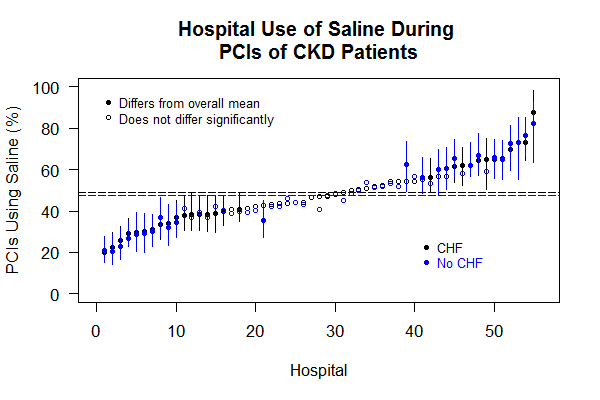


*The estimates are shown with 95% confidence intervals (CIs) for those that differ significantly from the system-wide average. Estimates are risk-adjusted for the following: age, sex, race (white, black, other), GFR, diabetes, lesion risk (at least 1 high vs all non-high), number of stents, year of PCI, and annual hospital PCI volume.*
